# Plasma proteomic profile of sulfur mustard exposed lung diseases patients using 2-dimensional gel electrophoresis

**DOI:** 10.1186/1559-0275-8-2

**Published:** 2011-01-07

**Authors:** Hossein Mehrani, Mostafa Ghanei, Jafar Aslani, Zahra Tabatabaei

**Affiliations:** 1 Laboratory of Proteomics, and Chemical Injuries Research Center, Baqiyatallah University of Medical Sciences, Tehran, Iran; 2 Department of Pulmonary Medicine and Chemical Injuries Research Center, Baqiyatallah University of Medical Sciences, Tehran, Iran

## Abstract

**Introduction:**

Sulfur mustard "bis (2-chlroethyl) sulphide" (SM) is a chemical warfare agent that remains a threat to human health. The aim of this study was to identify protein expression signature or biomarkers that reflect chronic lung damages induced by SM exposure.

**Methods:**

Prior to analysis, plasma was fractionated using ethanol precipitation. Using two dimensional SDS-PAGE; fractionated protein profiles of 20 healthy and 20 exposed patients with lung diseases were established. Selected protein spots were successfully identified with MALDI TOF MS/MS.

**Results:**

The results show that α1 haptoglobin isoforms were detected in plasma of the all lung disease patients but none of the healthy controls. Amyloid A1 isoforms was also detected in plasma of the lung disease patients but none of the healthy controls. Moreover, low molecular weight proteins were enriched in ethanol supernatant compared to ethanol precipitate.

**Conclusion:**

Our present results and previous studies suggest that ongoing tissue remodeling is involved in SM exposed lung damage patients. These finding might improve patient care and suitable therapies.

## Introduction

Sulfur mustard is a chemical warfare agent that remains a threat to human health.. More than lethality, SM causes debilitating effects that can leave an exposed individual incapacitated for days, months, or years. Lung injury is a common health problem after inhalation, which leads to chronic bronchitis and interstitial lung diseases [1]. The clinical picture of the poisoning is well known from the thousands of victims during World War I and the recent Iran-Iraq conflict. In the latter, sulfur mustard was heavily used and at the present time about 30,000 victims still suffer from late effects of the agent, such as chronic obstructive lung disease, lung fibrosis, recurrent corneal ulcer disease, and chronic conjunctivitis [2]. Late complications of mustard gas exposure and main clinical findings include; chronic bronchitis, bronchiectasis and bronchiolitis obliterans (BO) [3-5]. However, Clinical manifestation in lung disorders due to sulfur mustard is different from other lung diseases, due to the fact that mustard lung is not responsive to corticosteroids. There is no common consensus about the pathophysiological basis of chronic pulmonary disease caused by this chemical warfare agent [6].

Proteomics technologies can identify and quantify novel proteins in the plasma that can function as biomarkers of the presence or severity of disease states. In general, human plasma proteome profiling is challenging. Albumin is present at about 40 mg/ml and several other proteins are highly abundant including immunoglobulins (IgGs), transferrin and fibrinogen which typically constitute greater than 90% of total protein mass [7]. These abundant proteins may hinder the detection of low-abundant proteins that can be of specific interest in the search for biomarkers of disease [8]. However, it is the low abundant proteins that are most likely to be biologically relevant as the markers of a disease state. For analysis of low-abundant proteins in plasma, many strategies have been developed for the selective removal of albumin and other high-abundance proteins. Albumin can be removed by immune affinity columns chromatography [9], isoelectric trapping [10], heparin chromatography [11] and peptide affinity chromatography [12]. However, it is well known that albumin and other high-abundance proteins may also act as carrier or transport proteins and thus are likely to bind many species of interest, such as peptide hormones, cytokines, and chemokines.

There are wide-ranging interests in using the proteomics approach to define markers of lung disease. Although respiratory tract lesions represent the major disability after SM exposure, only a few studies have investigated the long term pathophysiology of SM induced respiratory damages, in particular their proteomes. We have recently examined the proteomics pattern in bronchoalveolar lavage (BAL) fluid of SM exposed patients and identified families of proteins whose expression is up or down regulated compared to healthy controls [13]. Plasma proteins and peptides are from almost every tissue and cell, and their change in quantity and quality is specific not only to the tissue affected by disease, but also to the disease process itself. In addition, plasma is the most easily accessible, less invasive, and widely collected sample.

We attempted to explore plasma proteomics patterns of these patients, using ethanol fractionation. Tow-dimensional gel electrophoresis was applied and followed by MALDI-TOF MS to look for new markers in the plasma of exposed patients which may help in further understanding the nature of long term effects of mustard gas. These finding might improve patient care and finding suitable therapies.

## Results

The plasma protein content of the patients and the controls are presented in Table 1. No significant differences were observed in plasma protein contents of patients and controls.

**Table 1:** Age and plasma protein concentrations of patients and control subjects ^a^

Variables	Healthy Controls	Severe Patients
Age (year)	40.1 ± 3.6	43.6 ± 2.8
Protein in plasma (mg/ml)	71.6 ± 3.1	72.4 ± 5.3

a. Data are mean ± SEM (n = 20 in each group).

**Figure 1:**
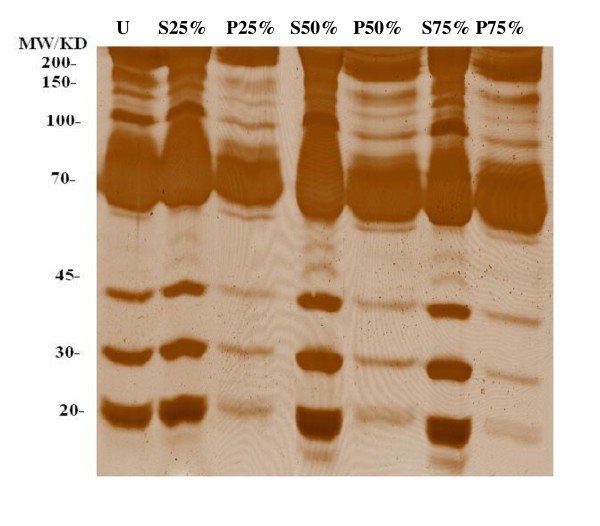
**SDS-PAGE of plasma proteins fractionated with different concentrations (v/v) of ethanol**. U represents plasma proteins before precipitation; S, ethanol supernatant; P, ethanol precipitate. Samples were analysed as described in the Method section and gels were stained using silver staining.

**Figure 2:**
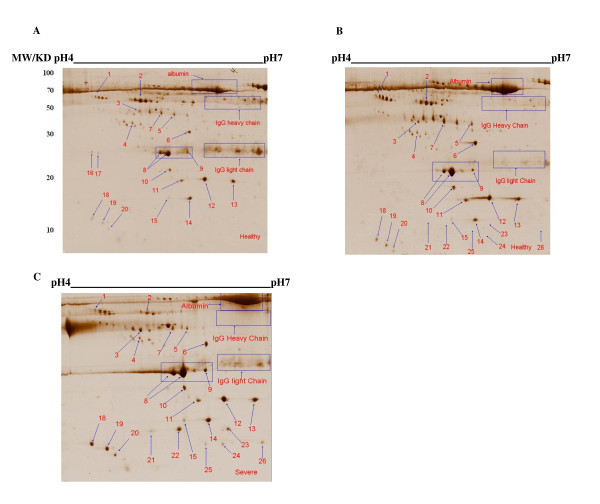
**Representative 2-DE protein patterns of plasma from healthy and severe lung diseases patients exposed to SM**. Plasma proteins were fractionated using 50% (v/v) ethanol fractionation. Sample preparation and 2-DE analysis were performed as described in the Materials and Methods section. Proteins (400 μg) were separated using linear IPG strips pH 4-7, followed by 15% SDS-PAGE and were detected by colloidal CBB staining. Each gel shows healthy control; ethanol precipitate (A); ethanol supernatant (B); and severe lung diseases patient; ethanol supernatant (C). Numbered spots indicate the MADI TOF MS/MS analysed proteins as listed in Table 3.

The ethanol fractionation was used to enrich low molecular weight proteins. As shown in Figure 1, most of the low molecular weight proteins were enriched in the ethanol supernatant rather than the precipitate. We found that 50% (v/v) ethanol was more efficient in fractionating low molecular weight proteins. To avoid any protein losses from the sample, we used both supernatants and precipitates of these fractions for 2-DE analysis. For the first dimension 24 cm IPG strips with pH values in the range of 4-7 were used. The proteins were resolved in homogeneous 15% acrylamide gels in the second dimension to obtain greater resolution for small proteins. About 300 spots in each colloidal CBB-stained gel can be visualized by ImageMaster software. Representative 2-DE images of plasma profiles from a healthy control for 50% (v/v) ethanol precipitate and ethanol supernatant are shown in Figure 2A and 2B respectively. Comparing the proteomics patterns of these two gels shows that both immunoglobulin heavy and light chains are separated in ethanol precipitate (Figure 2A) and albumin is distributed both in ethanol supernatant and precipitate (Figure 2A and 2B). Moreover, most of the small molecular weight proteins in the range of 15-50 kDa, have significantly (p < 0.05) higher spot volume and intensity in ethanol supernatant rather than precipitate (Table 2 and Figures. 2A and 2A).

**Table 2:** Comparison of spot volume and intensity in ethanol fractionated human plasma ^a^

Spot# b	Protein	Ethanol Supernatant		Ethanol Precipitate	
	**Name**	**% Volume**	**% intensity**	**% Volume**	**% intensity**

1	α2 HS glycoprotein	10 ± 1.4	23 ± 1.8**	8 ± 1.2	13 ± 1.6

2	α1-antitrypsin	160 ± 14	67 ± 6.4	170 ± 23	59 ± 4.7

3	Zinc α2 glycoprotein	36 ± 4.6**	46 ± 4.2**	12 ± 2.1	19 ± 4.8

4	SP40	20 ± 3.8	44 ± 2.7	21 ± 3.1	39 ± 3.2

5	Haptoglobin	36 ± 4.3**	51 ± 9.0**	19 ± 2.8	29 ± 2.5

6	Transthyretin	137 ± 19**	68 ± 5.6**	54 ± 13	33 ± 11

7	Apolipoprotein AIV	120 ± 11**	64 ± 8.3**	35 ± 7.8	37 ± 5.2

8	Apolipoprotein A1	140 ± 12	70 ± 4.1	121 ± 13	54 ± 5.9

8	Apolipoprotein A1	640 ± 23**	74 ± 8.2	405 ± 15	63 ± 6.2

9	Apolipoprotein A1	41 ± 3.0	59 ± 6.9	54 ± 4.7	36 ± 4.1

10	Retinol BP 4	53 ± 12**	58 ± 5.8*	27 ± 7.5	42 ± 5.5

11	Haptoglobin hp2α	73 ± 5.6**	56 ± 4.3**	41 ± 6.1	36 ± 4.2

12	Haptoglobin hp2α	215 ± 9.2**	70 ± 4.8	170 ± 8.5	62 ± 6.8

13	Haptoglobin hp2α	155 ± 8.4**	64 ± 6.8	91 ± 9.2	57 ± 6.8

14	Transthyretin	77 ± 5.8	56 ± 6.8	87 ± 9.2	54 ± 6.8

15	Transthyretin	10 ± 5.6	7.5 ± 6.8	11 ± 9.2	8.2 ± 6.8

18	Apolipoprotein CIII	123 ± 8.4**	58 ± 6.8 **	91 ± 9.2	27 ± 6.8

19	Apolipoprotein CIII	125 ± 8.4**	79 ± 3.4**	65 ± 7.8	25 ± 4.3

20	Apolipoprotein CII	35 ± 4.4**	24 ± 7.2**	11 ± 9.2	8.9 ± 6.8↑

Data are mean ± SEM of the % volume and intensity of protein spots on the 2D gels multiplied by 100. Significant difference between the samples were calculated using the Student's t-test and marked by * if p < 0.05, ** if p < 0.01.

a. Spot numbers correspond to those in Figures 2A and 2B

We analyzed the differences in the plasma protein patterns, comparing the gels of the diseased and healthy controls. The analysis of protein patterns of the plasma was focused on those protein spots which showed differences, comparing the patients and the controls. They were compared with Image Master 2-DE software and indicated only protein results in all cases (100%) with the same condition. As shown in Figures 2 B and 2C, twenty six protein spots were subjected to MALDI TOF MS analysis. All selected proteins and their isoforms were subsequently identified by PMF and MS/MS analysis. Table 3 lists the identities of the proteins and their isoforms which were analyzed in this experiment using MALDI TOF MS. Figures 2B and 2C shows the location of these protein spots in the 2-DE gels of a healthy controls and an exposed patients respectively. Volume and intensity of those protein spots which were only present in all patients' plasma but none of the healthy controls are shown in Table 4. Images from other healthy volunteers and patients were similar (data not shown).

**Table 3:** Analysis of tryptic digests with MALDI -TOF/TOF-MS (MS/MS)

Spot ^a ^ No	Protein name	Database ^b ^ id	MW/pI kDa	MS/MS ^c^	MALDI/MS ^d^	Score ^e ^ value	Coverage ^f ^(%)
1	α2 HS glycoprotein	gi156523970	39.3/5.43	4/4 [57, 101, 88, 71]	7/9	294	26
2	α1-antitrypsin	gi157831596	44.3/5.37	2/3 [58, 100]	9/13	301	47
3	Zinc α2 glycoprotein	gi4699583	31.6/5.70	4/7 [57, 36, 75, 58]	6/10	577	65
4	SP40	gi338305	36.7/5.74	4/4 [147, 65, 62]	7/12	170	43
5	Haptoglobin	gi3337390	38.2/6.14	3/5 [46, 117, 70]	6/12	209	31
6	Transthyretin	gi114318993	20.2/5.16	3/4 [102, 87, 32]	4/10	276	55
7	Apolipoprotein AIV	gi11957960	45.2/5.28	3/5 [61, 29, 42]	9/14	253	35
8	Apolipoprotein A1	gi178775	28.9/5.45	4/4 [54, 74, 57, 65]	7/16	482	73
9	Apolipoprotein A1	gi178775	28.9/5.45	4/5 [73, 37, 71, 61]	7/16	584	79
10	Retinol BP 4	gi18088326	23.0/5.76	3/4 [64, 125, 155]	6/10	472	58
11*	Haptoglobin hp2α	gi223976	41.7/6.23	2/3 [79, 36]	4/5	155	20
12*	Haptoglobin hp2α	gi296653	41.5/6.25	3/4 [92, 90, 53]	4/9	379	23
13*	Haptoglobin hp2α	gi296653	41.5/6.25	3/5 [42, 43, 30]	5/13	220	28
14	Transthyretin	gi4507725	15.9/5.52	3/4 [118, 175, 28]	5/10	476	73
15	Transthyretin	gi4507725	15.9/5.52	2/3 [114, 27]	3/8	216	65
16	IG J chain	gi114319027	19.6/5.24	3/4 [80, 43, 39]	4/4	199	25
17	IG J chain	gi114319027	19.6/5.24	2/3 [39, 28]	6/10	226	29
18	Apolipoprotein CIII	gi4557323	10.8/5.23	2/3 [105, 33]	3/3	181	34
19	Apolipoprotein CIII	gi4557323	10.8/5.23	2/3 [142, 58]	2/3	225	34
20	Apolipoprotein CII	gi4557323	11.2/5.42	2/3 [38, 24]	3/8	132	56
21*	Haptoglobin hp1α	gi3337390	38.2/6.14	2/4 [69, 27]	2/5	182	15
22*	Haptoglobin hp1α	gi3337390	38.2/5.28	2/2 [68, 59]	5/8	170	30
23*	Haptoglobin hp1α	gi337390	38.2/5.28	3/4 [63, 51, 53]	6/9	164	25
24	Serum Amyloid A1	gi40316910	13.5/6.28	3/5 [130, 110, 21]	4/7	129	63
25	Albumin fragment	gi19626079	22.4/6.2	3/8 [82, 85,103]	3/3	231	27
26	Serum Amyloid A1	gi40316910	13.5/6.28	3/3 [170, 130, 47]	6/9	181	80

a) Spot No. related to the annotation in Figure 2B and 2C. Number of peaks is given as matched to the protein/total number of SNAP algorithm selected peaks in the spectrum. Mass quality was assessed using transferrin and BSA as quality control internal standards. Score/coverage for transferrin and BSA was 421/50 and 216/29 respectively.

b) NRDB1 (6655203 protein sequence)

c) The column refers to the results of the MALDI-MS/MS analysis to the number and Mowse scores (in brackets) of assigned peptides.

d) The column refers to the results of the MALDI-MS PMF analysis of the number of assigned peptides to total number of measured peptide masses picked with SNAP algorithm.

e) MASCOT score value indicates the quality of database search results.

f) Sequence coverage, refers to the observed sequence coverage of the assigned protein

*. Molecular weight (MW) and p*I *are theoretical and * indicates proteins present on the gel at a MW or p*I *different to these values

**Table 4:** Spot volume and intensity in ethanol supernatant of lung disease patients ^a^

Spot# b	Protein Name	% Volume	% intensity
21	Haptoglobin hp1 α	33 ± 4.2	28 ± 5.2
22	Haptoglobin hp1 α	143 ± 7.6	78 ± 5.3
23	Haptoglobin hp1α	69 ± 6.7	62 ± 3.9
24	Serum Amyloid A1	28 ± 3.2	24 ± 4.3
25	Albumin fragment	31 ± 6.9	26 ± 6.4
26	Serum Amyloid A1	105 ± 9.3	69 ± 3.6

a. Spot numbers correspond to those in Figures 2C which were only present in all lung disease patients' plasma but none of the healthy controls.

Haptoglobin α1 chain isoforms (spots 21, 22 and 23) were only detected in the plasma of the severe lung diseases patients but were not detectable in healthy controls (Figure 2B and 2C). Furthermore, serum Amyloid A1 isoforms (spots 24 and 26) were only seen in the plasma of the patients but none of the healthy controls (Figure 2 B and 2C).

## Discussion

In this study we present plasma proteome analysis of SM exposed patients compared to the healthy controls. Human plasma and serum represent important biological materials for disease diagnosis. However, the wide dynamic range in protein concentrations remains a major challenge in the development of diagnostic assays. Human plasma albumin and the various forms of immunoglobulin represent the most abundant proteins in the plasma, constituting up to 80% of the total plasma proteins. The classical depletion strategy for albumin involves using hydrophobic dye Cibacron blue, a chlorotriazine dye which has high affinity for albumin [14]. As a group, the immunoglobulins represent the second most abundant proteins in the plasma or serum. It has been reported that albumin depletion may also remove some small low copy number proteins [15]. Therefore, in these experiments we used ethanol fractionation and found that this simple and low cost experimental procedure can be used for removing immunoglobulins and part of the albumin. Moreover, using 50% (v/v) fractionation, we showed that the ethanol supernatant contains all the protein spots that were found in the ethanol precipitate and recovers more small MW proteins.

We have identified some proteins that could give a novel insight into the pathogenesis of mustard lung. One of the main findings was different isoforms of haptoglobin in the plasma of severe lung disease patients but not in healthy controls. Haptoglobin is present in normal human plasma at a concentration range of 0.3-1.9 mg/ml, accounting for 0.4-2.6% of total plasma protein. Human haptoglobin is an inflammation-inducible plasma protein. It consists of 2 different types of α chains and a single type of β chain connected by disulfide bridges (β-α-α-β) giving 3 major phenotypes (Hp 1-1, Hp 2-1, Hp 2-2), the numbers 1 and 2 representing α1 (8.9 kDa) and α2 (16 kDa) chains, respectively. The β chain (40 kDa) is heavier than α chain and is identical in all Hp types [16-19].

Our results (Figures 2B and 2C) show that under our experimental conditions, the α2 chain (spots 11, 12, 13) is similarly expressed in the plasma of both experimental groups, but α1 chain (spots 21, 22, 23) is only expressed in the patients plasma samples but not in the healthy controls. In our recent study of BAL fluid proteomics patterns in SM exposed patients we also found that haptoglobin isoforms were significantly elevated in moderate and severe lung disease patients compared to mild and healthy controls [13]. Acute-phase proteins are induced shortly after exposure to triggering events such as inflammation, infection, and trauma, and are thought to be part of a general defense-response in injured tissue. But, these patients have been exposed to SM more than 20 years ago. It seems that an ongoing damage is occurring in lung of these patients. A recent proteomic study has also shown that levels of haptoglobin are elevated in BAL fluid in patients with mild asthma, and reported that haptoglobin may play a role in the differentiation of fibroblast progenitor cells, suggesting a novel role for haptoglobin in airway remodeling in patients with asthma [20]. Conversely Nishioka et al. showed that the serum concentration of haptoglobin was decreased during acute exacerbation in asthmatic children [21]. Haptoglobin isoforms and fragments are also elevated in plasma of severe acute respiratory syndrome (SARS) patients [22]. Haptoglobin plays a crucial role in defense against hemoglobin-induced oxidative stress by a mechanism thought to involve its high-affinity binding with hemoglobin and preventing iron release from hemoglobin. However, it has yet to be shown that haptoglobin itself is an antioxidant molecule [23]. Arredouani et al. [24] further described the role of haptoglobin affecting the immune system, showing that haptoglobin directly affects T-cells and suppress T-helper-cells through down-regulation of cytokine production.

We found that Amyloid A1 isoforms only detected in the plasma of severe lung diseases patients but not in healthy controls (Figure 2B and 2C). Similar results were reported for SARS patients [22]. Although serum Amyloid A1 is not as commonly used in human medicine as C- reactive protein (CRP), it is more sensitive to acute response than CRP [25,26]. Serum Amyloid A1 is an acute-phase protein that is induced, like CRP, by inflammatory mediators, including IL-6, IL-1β, and TNF-α, that rises in acute exacerbation of COPD [27]. Serum Amyloid A1 is secreted from the liver as the predominant apolipoprotein associated with plasma high density lipoprotein. We postulate that in our SM exposed patients ongoing tissue damage and repair (remodeling) is occurring which leads to an increase in acute phase reactant proteins such as haptoglobin and Amyloid A1.

In conclusion, this study complements our previous BAL fluid proteome analysis of patients exposed to SM gas which resulted in identification of number of differentially expressed proteins. To our knowledge this is the first study of plasma fluid proteome in SM exposed subjects. In this and our previous study the patterns of differentially expressed proteins identified in SM exposed patients are somewhat different from other lung diseases. It seems that SM exposed lung has an aberrant tissue remodeling which results in abnormal tissue architecture.

## Materials and Methods

### Chemicals

All chemicals used in these studies were analytical grade or equivalent and were obtained from Sigma (St. Louis, MO) unless otherwise noted. Milli Q water (18.2 MΩ) was used throughout.

### Patient population

According to the American Thoracic Society (ATS) classification and based on our spirometric and high resolution computed tomography (HRCT) findings, the patients were classified as severe lung diseases condition. Patients group included 20 male subjects. A group of 20 healthy age-matched male individuals was used as the control. This study was approved by the ethics committee of the research center of Baqiyatallah University of Medical Sciences, and informed consent was obtained from all patients and healthy controls. All participants were free to leave the study at will. The participant patients were suffering from pulmonary disorders due to previous exposure to a single high dose of SM gas during the Iran-Iraq conflict in 1987. Inclusion criteria were as follows: documented exposure to SM and documented diagnosis of chronic pulmonary disease due to mustard gas. Exclusion criteria for the patients and the control subjects were history of a chronic disease (tuberculosis, diabetes, hypertension, heart disease, hepatic diseases, etc.), resection of one or more lobes of the lungs, pneumonia and/or acute bronchitis, cigarette smoking or substance abuse. None of the patients or control subjects had a history of allergy or asthma. All patients and controls were in a stable condition and none of the participants had been administrated corticosteroids during the two-month period immediately preceding the studies.

### Sample collection

Fasting venous blood samples were collected in the morning (8-10 am). Blood samples were drawn into tubes containing K2EDTA and then immediately centrifuged at 1300 × g and 4°C for 10 min according to HUPO plasma proteomics project recommendation [28]. Supernatant was removed to a new tube and one tablet of complete protease inhibitor cocktail (Roche, Mannheim, and Germany) was added. Then, sample were promptly frozen in aliquots and stored at -80°C until used.

### Sample preparation

Plasma samples were thawed at room temperature and centrifuged at 10,000 × g for 10 min at 4°C. Then clear supernatants were transferred to new microfuge tubes for further processing. Total protein in plasma fluid was determined by the bicinchoninic acid (BCA) assay and employed bovine albumin as the standard (Pierce, Rockford, IL). The concentrations of proteins in plasma and different fractions were determined using a standard curve generated by the absorbance at 562 nm.

Following procedures for ethanol fractionation were applied. All steps were performed at 4°C. Plasma samples were diluted 1:1 (v/v) with Milli Q water in a new microfuge tube and equilibrated to 4°C by gentle mixing (continuously on a vortex mixer at a low setting) for 10 min. In new separate microfuge tubes containing 500 μl of diluted plasma samples, different volumes of cold ethanol were added and total volume was adjusted to 1000 μl with Milli Q water. Samples were incubated for an additional hour with gentle mixing on the vortex mixer and were centrifuged at 10,000 × *g *for 15 min at 4°C. Supernatants were removed to new tubes, pellets were briefly re-centrifuged and any residual supernatant was removed and added to the previous supernatant fraction. For electrophoresis, the supernatants were dialyzed against 50 Mm Tris-HCl (pH 7.4) overnight to remove ethanol.

### Polyacrylamide gel electrophoresis

For one dimensional SDS-PAGE analysis the collected ethanol supernatants and pellets were reconstituted in 50 mM Tris-HCl (pH 6.8), 2% (w/v) SDS, 0.1% (w/v) bromophenol blue and 10% (v/v) glycerol. Proteins were separated by mini 1-DE (10% T resolving gels with 4% stacking gels; 10 μg protein/lane). Gels were electrophoresed at 30 V for 30 min and then at 50 mA per gel for 45 min, and stained using Vorum silver staining procedure as described recently

[29]. For 2-DE analysis dialyzed ethanol supernatants and pellets were reconstituted in buffer consisting of 7 M urea, 1 M thiourea, 20 mM Tris, pH 7.5, 4% (w/v) 3-[(cholamidopropyl) dimethylamino]-1-propanesulfonate (CHAPS), and centrifuged at 10,000 × g at room temperature for 5 min. Supernatants were taken for 2-DE gel analysis. Protein concentration in the recovered samples was determined using a modification of the method as described by Bradford [30].

Two dimensional gels were analysed using 400 μg proteins per 24-cm linear IPG strips pH 4-7 (Bio Rad) as described previously [13]. IPG strips were reduced and alkylated, and then proteins were separated in the second dimension on homogeneous 15% polyacrylamide gels in an Ettan DALTsix electrophoresis unit (GE healthcare, Uppsala, Sweden). IPG strips were placed on the top of polyacrylamide gels (1 × 200 × 260 mm) and sealed with a solution of 1% (w/v) agarose containing a trace of bromophenol blue. Gels were run in a running buffer, containing 25 mM Tris, 192 mM glycine, and 0.1% (w/v) SDS at 2W/gel for 45 min, followed by 6W/gel at 20°C until the bromophenol blue had migrated to the bottom of the gel. Gels were fixed overnight in acetic acid, methanol, water, 10:45:45 (v/v) and stained with colloidal Coomassie brilliant blue (CBB) G-250 as described previously [13].

### Gel analysis and protein identification

Analysis of spot patterns was performed using ImageMaster 2D Platinum software (version 6.0; GE Healthcare). CBB stained gels were scanned in transmission scan mode using Bio-Rad GS-800 calibrated densitometer at a resolution of 600 dots per square inch (dpi). The scanned gels were saved as TIF images for subsequent analysis. Protein spots were detected automatically. Manual spot editing or deleting (of artifacts) was performed when necessary. The spots intensity, volume, or saliency was adjusted in the preview mode using Image master software. Furthermore spots on all gels were visually carefully inspected for inappropriate matching, staining artifacts, or bad spot detection and conserved for analysis. To measure the volume and intensity of protein spots on CBB-stained gels the volume and intensity of each spot was divided by the total volume or intensity of all spots of the same gel. Since this method of normalization produces extremely small values, the result was multiplied by a scaling factor of 100, which produced spot percentage volumes or intensity. Only those spots that were reproducibly and statistically significant (p < 0.05) in intensity or volume were used for analysis.

For mass analysis the protein spots of interest were aseptically removed under a laminar flow hood. Processing of gel plugs, trypsin digestion and MALDI TOF MS/MS analysis using a Bruker Autoflex III MALDI TOF/TOF instrument (Alphalyse, Denmark) and database searching was performed as described previously [13]. The MS and MS/MS spectra were combined and used for a database search using the Mascot software (Matrix science, version 2.2.03) for database searches with the selection of following criteria: Database search program: NRDB1 (6655203 protein sequence), species of origin *Homo sapience*, peptide ion mass tolerance 60 ppm,, MS/MS tolerance 0.2 Da, peptide cut-off of 25, and digestion by trypsin allowing for no more than one missed cleavage. The accuracy of mass detection was MH+ of 0.01 assuming possibility of modification of cysteine by acrylamide and oxidation of methionine. Proteins identification was based on a combination of the peptide mass fingerprint (PMF) with peptide masses, and several MS/MS spectra of selected peptides in each MALDI MS spectrum. The protein and protein isoforms shown on the identification list is based purely on the human protein database accession that gives the highest Mascot score. To the extent that the different sequence isoforms are represented in individual database accession numbers, these have been considered in the database search. Other modifications and isoforms are not considered in this analysis. The positive protein identification was based on a probability-scoring algorithm (http://www.matrixscience.com) and the 95% confidence level for positive identification was a score = 80

### Statistical analysis

All data are representative of at least three independent experiments with twenty individuals in each group and are expressed as means ± standard error of the mean (SEM).	Comparisons between two groups were performed using unpaired Student's t-test. The criterion for statistical significance was p < 0.05 for all comparisons.

## Abbreviations

2-DE: 2-dimensional electrophoresis; Apo A1: Apolipoprotein A1; ATS: American Thoracic Society; BAL: bronchoalveolar lavage; BCA: bicinchoninic acid; BO: bronchiolitis obliterans; CBB: Coomassie brilliant blue; CHAPS: 3-[(cholamidopropyl) dimethylamino]-1-propanesulfonate; COPD: chronic obstructive pulmonary diseases; CRP: C- reactive protein; DTT: dithiothreitol; HRCT: high resolution computed tomography; IPG: immobilized pH gradient; K2EDTA: di-potassium ethylene di-amine tetra acetic acid; MALDI TOF: matrix assisted laser desorption ionization time of flight; MS: mass spectrometry; MS/MS: tandem mass spectrometry; PMF: peptide mass fingerprint; SARS: severe acute respiratory syndrome; SDS-PAGE: sodium dodecyl sulfate polyacrylamide gel electrophoresis; SM: sulfur mustard.

## Competing interests

The authors declare that they have no competing interests.

## Authors' contributions

HM carried out study design, analysis of proteomics profile, Data mining and drafted and managed the manuscript. MG participated in the design of study and patient handling. JA participated in the patient handling and funding management. ZT participated in the experimental laboratory work on proteomic profiling. All authors read and approved the final manuscript.
